# Activity of the Substantia Nigra Pars Reticulata during Saccade Adaptation

**DOI:** 10.1523/ENEURO.0092-23.2023

**Published:** 2023-09-11

**Authors:** Yoshiko Kojima, Daisuke Koketsu, Paul J. May

**Affiliations:** 1 Department of Otolaryngology–Head and Neck Surgery, Washington National Primate Research Center, University of Washington, Seattle, WA 98195; 2 Division of System Neurophysiology, National Institute for Physiological Sciences, Okazaki 444-8585, Japan; 3 Department of Advanced Biomedical Education, University of Mississippi Medical Center, Jackson, MS 39216

**Keywords:** adaptation, learning, monkey, saccade

## Abstract

When movements become inaccurate, the resultant error induces motor adaptation to improve accuracy. This error-based motor learning is regarded as a cerebellar function. However, the influence of the other brain areas on adaptation is poorly understood. During saccade adaptation, a type of error-based motor learning, the superior colliculus (SC) sends a postsaccadic error signal to the cerebellum to drive adaptation. Since the SC is directly inhibited by the substantia nigra pars reticulata (SNr), we hypothesized that the SNr might influence saccade adaptation by affecting the SC error signal. In fact, previous studies indicated that the SNr encodes motivation and motivation influences saccade adaptation. In this study, we first established that the SNr projects to the rostral SC, where small error signals are generated, in nonhuman primates. Then, we examined SNr activity while the animal underwent adaptation. SNr neurons paused their activity in association with the error. This pause was shallower and delayed compared with those of no-error trial saccades. The pause at the end of the adaptation was shallower and delayed compared with that at the beginning of the adaptation. The change in the intertrial interval, an indicator of motivation, and adaptation speed had a positive correlation with the changes in the error-related pause. These results suggest that (1) the SNr exhibits a unique activity pattern during the error interval; (2) SNr activity increases during adaptation, consistent with the decrease in SC activity; and (3) motivational decay during the adaptation session might increase SNr activity and influence the adaptation speed.

## Significance Statement

Movements impaired because of stroke, injury, or aging recover gradually. To repair an inaccurate movement, the brain measures the error of the movement and changes the movement to minimize the error. This process is called motor adaptation. Although it has been established that the cerebellum plays an essential role in this adaptation, the influence of the other brain areas on the adaptation process is not well defined. The observation that motivation levels influence adaptation led us to suspect that the basal ganglia may influence adaptation. Here, we reveal the substantia nigra pars reticulata (SNr) activity changes during saccade adaptation in monkeys and suggest how the basal ganglia influence the error signal.

## Introduction

For goal-directed movements, the brain detects any postmovement error, which is the difference between the desired movement and the actual movement, and adjusts subsequent movements to reduce the error. This process is called motor adaptation. Saccades are a useful model to study the underlying neural mechanism of the postmovement error adaptation process because: (1) their neural substrate has been well studied (Scudder et al., 2002) and (2) there is an established paradigm to induce adaptation by the behavioral imposition of an apparent saccade error (F.R. Robinson et al., 2003; Kojima et al., 2015; Kojima and Soetedjo, 2017a). In this paradigm, the eye position at the end of the saccade is detected and the target is quickly displaced relative to that position, so that the saccade appears to be in constant error. Depending on the error direction, the paradigm can induce adaptation to decrease or increase the saccade amplitude or change the saccade direction.

Various studies have indicated that amplitude decrease saccade adaptation, the most commonly studied saccade adaptation, relies on plastic neuronal changes in the cerebellar cortex (Catz et al., 2008; Kojima et al., 2010). The error signal that drives adaptation arrives over the climbing fiber pathway to the oculomotor vermis (Catz et al., 2005; Soetedjo and Fuchs, 2006; Soetedjo et al., 2008a,b). This climbing fiber pathway originates from the part of the inferior olive that receives input from the superior colliculus (SC; Harting, 1977; Yamada and Noda, 1987). SC activity corresponding to the postsaccadic visual error has been recorded and found to decrease during saccade adaptation trials (Kojima and Soetedjo, 2017a). This suggests that the visual error sensitivity in the SC decreases as the saccade adaptation trials proceed. A logical next step is to examine inputs to the SC during adaptation to reveal how SC error sensitivity is modulated.

In the substantia nigra pars reticulata (SNr), an output nucleus of the basal ganglia that directly inhibits the SC, nigrotectal cells pause their tonic activity for saccades, and this pause is correlated to the motivation state (Hikosaka et al., 2000). Pharmacological inactivation of the SNr facilitates saccade amplitude decrease adaptation (Kojima and May, 2021), indicating that while the SNr is not required to induce adaptation, it does influence saccade adaptation. The present experiments were directed at determining the role of the SNr in saccade adaptation by recording SNr activity during this adaptation paradigm.

The first question we asked in this study is whether the SNr activity changes during the adaptation session. The second question we asked is whether the SNr activity increases or decreases as the number of trials in the session accrues. If SNr activity influences the SC error activity, then SNr activity during the error interval should increase during saccade adaptation. At the outset, we specifically confirmed that the SNr projects to the rostral SC, as this region responds to small vector visual stimuli and the amplitude of the error is small in this experiment. The last question we asked is what signals, such as motivation, might cause the changes in the SNr activity over the adaptation trials. Saccade amplitude is not the only change that occurs during the adaptation session. Other parameters that change include, saccade latency, trial-by-trial interval, adaptation speed and trial number. This suggests that many signals change during adaptation. Although these signals are difficult to isolate and we cannot examine cause and effect in this experiment, we did examine the correlation between the SNr activity changes and the changes in other signals that were available in our data.

## Materials and Methods

All experiments were performed in accordance with the Guide for the Care and Use of Laboratory Animals and exceeded the minimal requirements recommended by the Institute of Laboratory Animal Resources and the Association for Assessment and Accreditation of Laboratory Animal Care International. All the procedures were evaluated and approved by the local Animal Care and Use Committee of the University.

### Surgery and training

Three male Macaca mulatta monkeys (E, Z, and N) participated in this study. We implanted each monkey with fixtures to prevent head movements, a scleral search coil (Judge et al., 1980) to measure eye position in space, two recording chambers that were aimed at the substantia nigra pars reticulata (SNr) on each side, and a recording chamber that was centered on the sagittal fissure to allow both superior colliculi to be targeted.

After the monkeys had recovered from the surgery, we trained them to track a small visual target in a dimly lit, sound-attenuating booth. The target was a 0.3° laser spot projected onto a tangent screen via two computer-controlled orthogonal mirror galvanometers. The screen was 65 cm from the monkey’s eyes. The monkey sat in a primate chair with its head restrained. We measured eye position with the electromagnetic search coil method (Fuchs and Robinson, 1966). We rewarded the monkeys with applesauce for keeping their gaze within a ±2° window around the horizontal and vertical position of the target spot for at least 0.5 s. Once they had learned to fixate the target spot, we trained them to make visually-guided saccades to a stepping spot that moved to random locations on the tangent screen within a ±18° radius of straight-ahead. We also trained them to make delayed saccades. In this paradigm, the monkey must saccade to a new spot only after the fixation spot is extinguished (0.5 s overlap). We delivered the applesauce reward (∼0.16 ml per dollop, ∼200 ml/h) by a pump (Masterflex tubing pump, Cole-Parmer) every 2 s regardless of the amplitude, direction, or timing of the saccade, as long as it landed within the ±2° window surrounding the target. The targeting saccade was required to occur within 0.6 s of the target step and the subsequent fixation had to be maintained for at least 0.3 s for a reward to be given.

### Experimental procedures

After the monkey reliably tracked the jumping target spot, we started recording experiments to find the SNr region where neurons exhibit tonic activity during fixation and paused for both visual stimuli and saccades in the contraversive direction (Hikosaka and Wurtz, 1983). As previously reported (Handel and Glimcher, 1999), we also found other activity patterns, but we focused only on those neurons with the pattern just described. In total, we recorded activity in the 58 units in two monkeys (43 neurons from monkey E and 15 from monkey Z).

**Figure 1.**
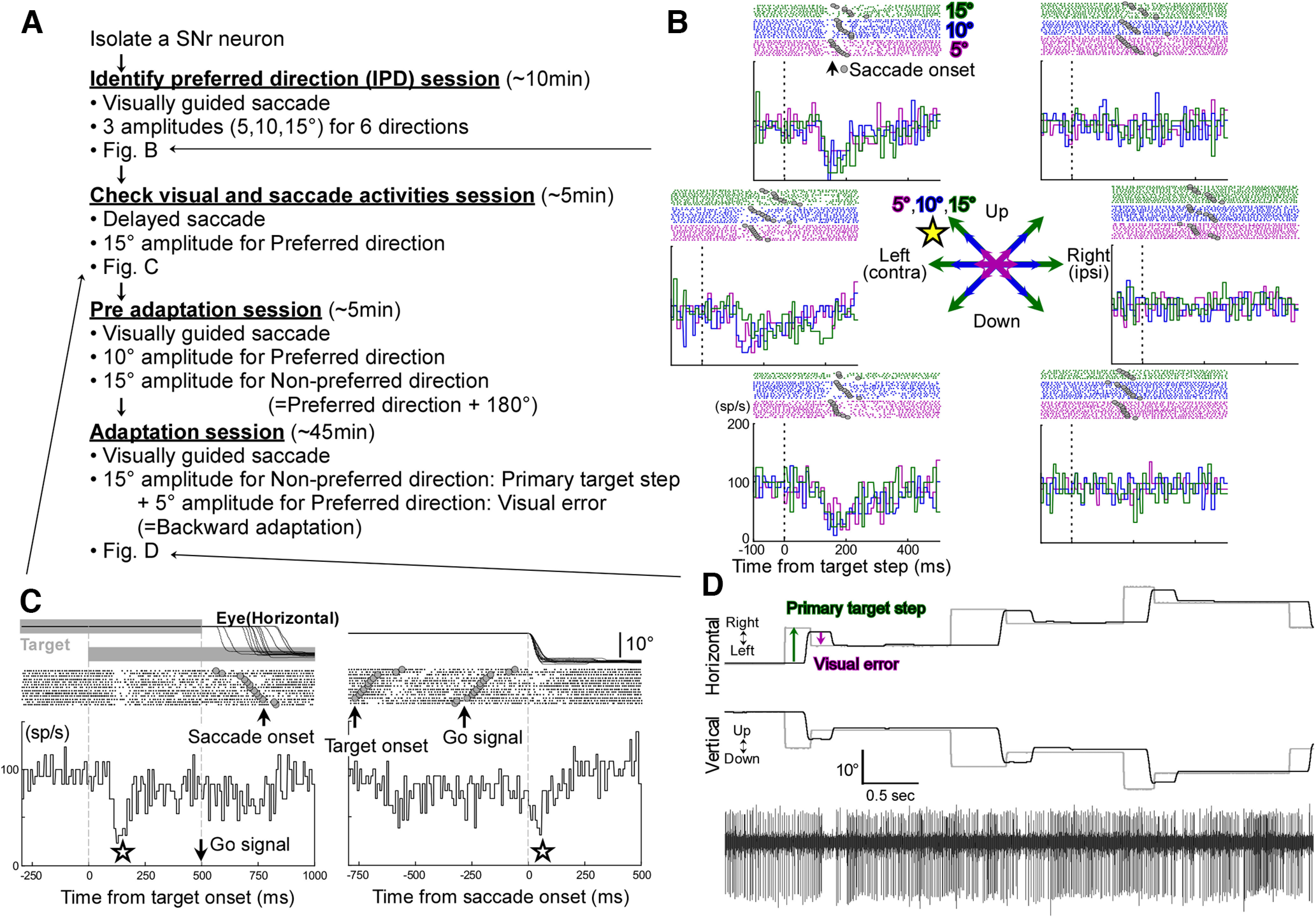
Experimental procedure and the response of a typical right SNr neuron (neuron #10, monkey E). ***A***, A diagram of the procedure. ***B***, Visually-guided saccades to three target amplitudes (5°, 10°, 15°, indicated by different colors) in six directions. The neuron pauses for all contraversive directions and all three amplitudes. We selected left-up as this neuron’s preferred direction (star mark). ***C***, Delayed saccade task. Data are aligned on target onset (left panel) and saccade onset (right panel). The target stepped 15° in the neuron’s preferred direction (left up). This neuron exhibited a pause that began at the target onset (star mark, left panel) and for the saccade to target (star mark, right panel). Responses were sorted by saccade latency. ***D***, A representative recording of neuronal activity, along with eye and target positions, during adaptation (lower panel). A 5° postsaccadic error in the neuron’s preferred direction was provided after every primary saccade in their nonpreferred direction (upper panel).

We used glass coated tungsten micro-electrodes (Alpha-Omega) guided by a 21-gauge hypodermic cannula. Once we isolated an SNr neuron, we tested visually-guided saccades to three target amplitudes (5°, 10°, 15°) in six directions (right, 45° right up, 45° left up, left, 45° left down, and 45° right down; randomly interleaved) to identify the neuron’s preferred direction based on where the pause was the widest and deepest [Fig. 1*A*, identify preferred direction (IPD) session]. As reported previously (Hikosaka and Wurtz, 1983; Hikosaka et al., 2000), SNr neurons paused for visual stimuli and saccades in the direction contraversive to the recording side. Figure 1*B* shows a representative recording of the activity associated with visually-guided saccades for three target amplitudes in six target directions aligned on the target step (vertical dashed lines). This neuron, recorded in the right SNr, exhibited the typical broad response field, pausing for all three tested amplitudes (5°, 10°, 15°) in the three tested leftward (contraversive) directions (45° left-up, left, and 45° left-down). We designated the left-up direction as this neuron’s preferred direction because the pause occurring after the target step was the deepest and widest for all three amplitudes along this direction.

For the neuron’s preferred direction, we tested delayed saccades to 15° target steps to differentiate between the pauses for visual stimuli and those for saccades (Fig. 1*A*, check visual and saccade activity session). In this task, the time between the target step onset and the following saccade onset is long enough to dissociate the pause for the visual stimuli from the pause for the saccade. Consistent with the foundational study of SNr neurons (Hikosaka and Wurtz, 1983), there is a clear pause after target onset (Fig. 1*C*, left panel, first vertical dashed line) and also a pause at saccade onset (Fig. 1*C*, right panel, vertical dashed line).

Next, we tested visually-guided saccades to 10° target steps for the neuron’s preferred direction and 15° target steps for the neuron’s nonpreferred (180° opposite) direction as a preadaptation session for ∼ 5 min (Fig. 1*A*, preadaptation session).

Finally, we started the adaptation paradigm [intrasaccadic target step (ISS); Fig. 1*A*, adaptation session]. Because the visual error at the end of the saccade drives saccade adaptation, we examined visual errors in the neuron’s preferred direction. For example, if the preferred direction for a neuron was left-up, the ISS was always left-up during the experiments (Fig. 1*D*). Consequently, to induce amplitude decrease adaptation, the primary saccades were directed in the nonpreferred direction. Note that the stimulus presentation for saccade decrease adaptation has been shown to appear to the viewer as if the saccade was too large, not that the target was shifted, because of the lag in the visual system and the fact that visual sensory information is suppressed during and immediately after the saccade (Frens and van Opstal, 1994; Wurtz, 2008; Wurtz et al., 2011). Thus, the primary saccade will appear to the monkey to have overshot for amplitude decrease adaptation. Under these conditions, the primary saccade is followed by a corrective saccade that allows the eye to obtain the target. To keep the vector of the postsaccadic visual error constant at 5° during the entire adaptation session, we used a modified version of the conventional McLaughlin paradigm (McLaughlin, 1967; F.R. Robinson et al., 2003; Kojima et al., 2015; Kojima and Soetedjo, 2017a; Kojima and May, 2021). As the monkey made a saccade toward the target, we measured the eye position at the end of the saccade (determined when eye velocity fell to 20°/s), and immediately stepped the target by 5° from the landing position. Consequently, the amplitude of corrective saccades also remained constant at ∼5° (across all adaptation experiments, the mean difference in corrective saccade amplitude of the first and last 50 adaptation trials was 0.069 ± 0.31°; Table 1). To begin the next trial, the target stepped from the displaced target position. Therefore, the target start position of each new trial was random. This can be seen in Figure 1*D*. If this target movement is repeated for several hundred trials, a gradual amplitude decrease adaptation occurs. Across the 58 amplitude decrease adaptation experiments, the mean number of trials was 956.1 ± 568, and the mean amplitude change of the primary saccade to the 15° target step was 2.30 ± 1.1° (= first 50–last 50 adaptation trials; Table 1). To move the target within the target step area (within a ±18° radius of straight-ahead), the target also stepped in the preferred direction to elicit a 10° primary saccade with a 5° visual error that was also directed to the preferred direction. This arrangement induces amplitude increase adaptation. However, the mean amplitude change was only 0.81 ± 0.55° across the 58 amplitude increase adaptations because the mean number of trials was only 418.8 ± 306.3. This was because of the fact that when the primary and corrective saccades are in the same direction, as for amplitude increase adaptation, there is a more limited target step area (within a ±18° radius of straight-ahead). The program randomly chose the target step direction (preferred or nonpreferred direction) when the space was available for both directions within the area. However, when a target step toward one direction would cause the target to fall out of the area, then the program always chooses the other direction, to keep the target within the area. Furthermore, each SNr pauses for the contraversive (preferred) direction, but displays no phasic activity for the ipsiversive (nonpreferred) direction. The pause in SNr activity for the primary saccades (preferred direction) interfered with the pause for the postsaccadic visual errors (preferred direction) in the amplitude increase paradigm. In contrast, there was no interference for the amplitude decrease adaptation because the primary saccade is in the nonpreferred direction. For these reasons, we excluded the amplitude increase adaptation data from this analysis.

**Table 1:** Summary of the experimental condition and behavioral effects across 58 experiments

			Across 58 experiments	
			Mean ± SD	*p*
	Total adaptation trial number		956.1 ± 568	
	Total adaptation time (min)		46.7 ± 25.8	
Change (Last50-First50)	Primary saccade	Amplitude (°)	−2.3 ± 1.1	4.50 × 10^−6^
		Latency (ms)	22.7 ± 18.0	2.00 × 10^−5^
	Corrective saccade	Amplitude (°)	−0.069 ± 0.31	0.058
		Latency (ms)	35.3 ± 21.6	1.50 × 10^−7^
	Visual error	Amplitude (°)	0.0033 ± 0.16	0.62
	Trial-by-trial amplitude change (°/trial)		0.030 ± 0.047	5.40 × 10^−5^
	Intertrial interval (s)		0.107 ± 1.24	0.047
Adaptation course	Saccade amplitude = a × exp (b × TrialNumber) + c	a	−2.9 ± 1.3	
		b	0.011 ± 0.048	
		c	10.7 ± 1.4	
		sse	1093.5 ± 815.8	
		*r* ^2^	0.29 ± 0.16	

### Neuroanatomy

**Figure 2.**
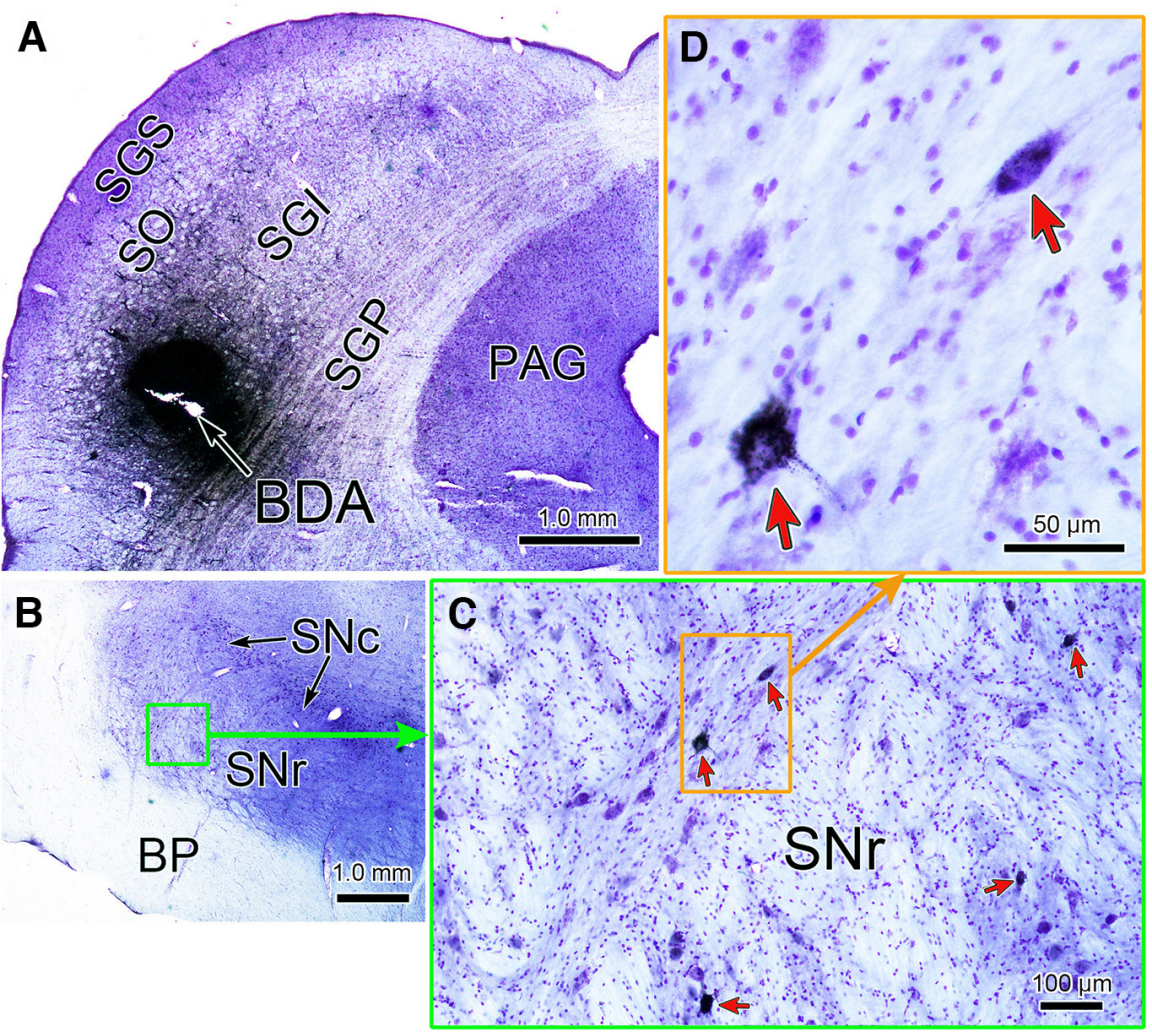
Labeled nigrotectal neurons after a BDA injection into the rostral SC. ***A***, BDA injection site in the intermediate gray layer (SGI) of the left SC of monkey N at a site that yielded 4.1° saccades on stimulation. ***B***, Section through the left SNr showing region with labeled cells. The area in the green rectangle is enlarged in ***C*** to show the retrogradely labeled cells (red arrows) in the nucleus. Two examples, contained in the orange box are shown at higher magnification in ***D***. Note the black particulate label in cells. BDA = biotinylated dextran amine, BP = basis pedunculus, PAG = periaqueductal gray, SNc = substantia nigra pars compacta, SNr = substantia nigra pars reticulata, SGI = intermediate gray layer, SGP = deep gray layer, SGS = superficial gray layer, SO = stratum opticum.

In two of the three monkeys (E and N), we injected biotinylated dextran amine (BDA; 10,000 MW, Invitrogen, Thermo Fisher Scientific) into the rostral superior colliculus (Fig. 2*A*) through a 35-gauge stainless steel tube. The tube was insulated by epoxylite except for at its beveled tip to allow electrical stimulation. To prepare for the injection, we plotted the topographic map of the rostral SC (D.A. Robinson, 1972; Sparks and Mays, 1980; Munoz and Wurtz, 1995) by recording unit activity and using electrical stimulation (50 μA, 500 Hz, 50 ms trains of 0.1 ms cathodal pulses) with conventional electrodes. On the day preceding each injection, we made electrode penetrations of this type into the SC to identify the optimal vector of the chosen locus (Sparks and Mays, 1980; Munoz and Wurtz, 1995; Soetedjo et al., 2002a, b; Kojima and Soetedjo, 2017a). On the day of the injection, we advanced the tip of the injectrode toward the same site until we heard neuronal bursts related to pseudo-random (in direction and size) target steps and/or the targeting saccades (Kojima and Soetedjo, 2018). We then stimulated to evoke saccades and took the site’s preferred vector as the average vector of 5 evoked saccades. For monkey N (Fig. 2, left SC), the stimulation evoked a 4.1° saccade in a 337° direction (right and down). For monkey E (right SC), the stimulation evoked a 1.7° saccade in a 144° direction (left and up). In each animal, we injected 120 nl of 10% BDA by using brief pulses of air pressure (PV830 Pneumatic PicoPump, WPI).

Twenty-two days after the BDA injection, the two animals were sedated with ketamine (20 mg/kg), deeply anesthetized with isoflurane gas, administered heparin (1000units/kg), and perfused with 4% paraformaldehyde in 0.1 m, pH 7.2 phosphate buffer (PB). Serial sections of the brain were cut in the frontal plane using a Vibratome (Leica) at a thickness of 100 μm. To visualize the BDA, sections were first incubated in avidin conjugated to horseradish peroxidase diluted 1:500 in PB with 0.03% Triton X-100 for 18 h at 4°C. They were then reacted with the chromagen diaminobenzidine HCl (DAB). This process consisted of an incubation in 0.5% DAB in 0.1 m, pH 7.2 PB, with 0.005% cobalt chloride and 0.01% nickel ammonium sulfate. The reaction was catalyzed by the addition of 0.005% hydrogen peroxide and stopped by rinses in PB. Sections were then mounted onto gelatinized slides, counterstained with cresyl violet, dehydrated, cleared and coverslipped. Images of the labeling were taken with a Nikon Eclipse E600 microscope equipped with a DS-Ri1 digital camera. Nikon Elements software controlled the image acquisition and Adobe Photoshop was used to adjust the color and contrast of the images to more closely match the appearance of the material as viewed through the eyepieces.

### Data analysis

We digitized eye and target position signals at 1 kHz and sampled unit activity at 50 kHz using Power1401 data acquisition/controller hardware (Cambridge Electronic Design). Data were saved to a hard drive for later analysis. A custom program running in Spike2 (Cambridge Electronic Design) controlled target movement and the monkey’s reward via the Power1401 hardware.

Another custom program running in Spike2 analyzed the saved data. It detected the occurrence of a saccade when eye velocity exceeded 75°/s within 70–800 ms after a target jump and marked saccade onset and end when the vector’s eye velocity exceeded or fell below 20°/s, respectively. The program measured saccade amplitude, peak velocity, and duration, as well as the target distance for each saccade. A voltage threshold detected each action potential and the program saved its time of occurrence. The marked events were carefully checked by eye and if a unit’s isolation became problematic (e.g., the spikes were contaminated by activity from the other units, the spikes of the unit became too small, etc.), we discarded it. All 58 neurons used in this study were well isolated during the entire adaptation session. The saccade attributes, target positions, and action potential times were exported to MATLAB (MathWorks). Saccades whose initial eye positions differed from initial target positions by >5° were not analyzed. To analyze SNr activity, we created a spike density function (SDF) by replacing each action potential with a Gaussian function (12 ms SD; Basso and Wurtz, 2002; Basso et al., 2005) centered on the time of spike occurrence.

**Figure 3.**
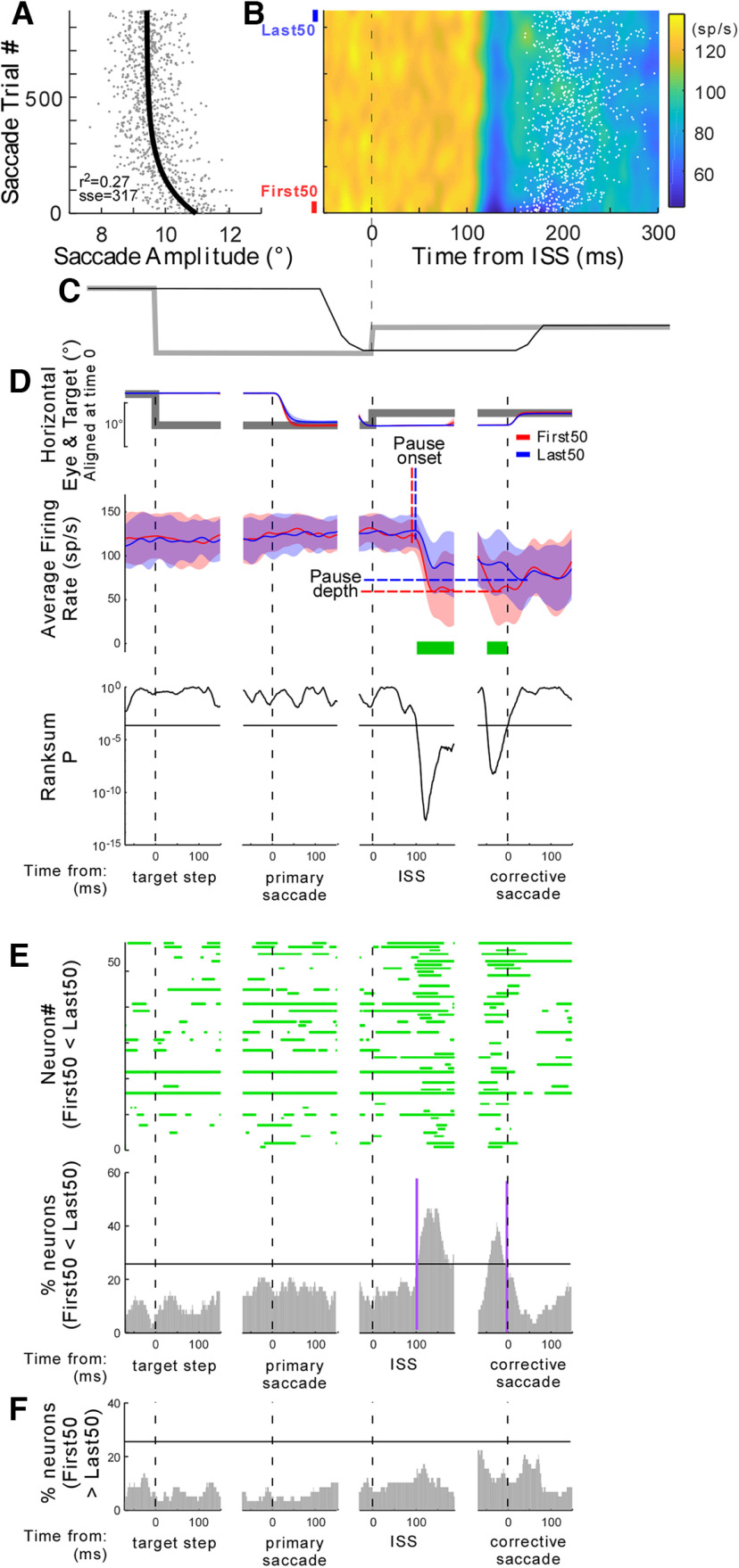
Activity of a representative neuron in the left SNr (neuron #43, monkey E) during gain decrease adaptation. ***A***, Decrease in primary saccade amplitude with saccade trial number (bottom to top). Gray dots represent individual primary saccades; black line is an exponential fit. ***B***, Colorized firing rate during adaptation aligned on the intrasaccadic step (ISS) at the end of the primary saccade (time = 0). The white dots represent the corrective saccade onsets of each trial. The first and last 50 saccades are marked by red and blue lines (on left), respectively. ***C***, A representative trial that shows the target (gray) and eye movement (black) during adaptation aligned on the data in ***B***. ***D***, Average firing rates associated with the first (red) and last (blue) 50 saccades. Data are aligned on primary target step time (left), primary saccade onset (second from left), ISS (second from right), or corrective saccade onset (right). Top panel, Horizontal eye (red and blue lines) and target (gray line) position. Middle panel, Average firing rate associated with the first (red) and last (blue) 50 saccades. Red and blue vertical and horizontal broken lines: pause onset and pause depth associated with the first (red) and last (blue) 50 saccades, respectively. The tinted shadows indicate 1 SD. Bottom panel, *p* values of Wilcoxon rank-sum tests for every millisecond from the comparison of average firing rates for the first and last 50 saccades in the middle panel. Horizontal lines are significant level (*p* = 0.05 with Bonferroni correction). Green lines at the bottom of the middle panel indicate the time of significant difference. ***E***, Summary of recorded neuron responses. Top panel, Significant time of these 58 recorded neurons. Bottom panel, Histogram (significant time histogram, STH; 1 bin = 1 ms) of the percentage of recorded neurons (% neurons) that exhibited significantly greater activity in the last 50 saccades than in the first 50 saccades. The horizontal line indicates the threshold for significance (25.8%, which is the mean ± 1 SD of all bins). For each monkey, see Extended Data Figure 3-1. ***F***, STH from the same records, showing neurons that exhibited significantly weaker activity in the last 50 saccades than the first 50 saccades.

To analyze the changes in saccade amplitude during adaptation, we plotted it against the saccade trial number (Fig. 3*A*). To document the course of adaptation, we fitted the results with an exponential function because it is the consensus fit in the literature (Straube et al., 1997; Hopp and Fuchs, 2004; Kojima et al., 2015):

Saccade amplitude = a × exp(b × TrialNumber) + c.

10.1523/ENEURO.0092-23.2023.f3-1 Extended Data Figure 3-1 STH of the percentage of recorded neurons (% neurons) that exhibited significantly greater activity in the last 50 saccades than in the first 50 saccades (Fig. 3*E*, bottom) for each monkey. The horizontal line indicates the threshold for significance (23.7% for monkey E and 37.8% for monkey Z, which is the mean ± 1 SD of all bins). Download Figure 3-1, TIF file.

Here, a, b, and c are coefficient parameters. The mean sum of squares due to error (sse) and *r*^2^ of the fit across 58 experiments were 1093.5 ± 815.8° and 0.29 ± 0.16°, respectively (Table 1).

To visualize the neuronal discharge, we smoothed the SDFs across trial numbers along the vertical axis by a moving average with a span the size of a bin width (25 trials) and presented the data as a surface plot (Fig. 3*B*).

To visualize the change in the discharge patterns, we compared the average SDF of the first and last 50 adaptation trials (Fig. 3*D*). We analyzed four intervals. (1) For baseline activity (before the primary target step) and visual activity after the primary target step, we aligned the SDF on the primary target step onset and analyzed 70 ms before to 150 ms after the target step (total: 220 ms). (2) For primary saccade activity, the SDF was aligned on the primary saccade onset and analyzed 70 ms before to 150 ms after the saccade onset (total: 220 ms). (3) For postsaccadic visual error activity, the SDF was aligned on the ISS and analyzed 30 ms before to 190 ms after the ISS (total: 220 ms). (4) For corrective saccade activity, the SDF was aligned on the corrective saccade onset and analyzed 70 ms before to 150 ms after the corrective saccade onset (total: 220 ms). To determine when the SDF for the first and last 50 saccades were different in each interval, we performed Wilcoxon rank-sum tests, using a 2-ms time slice with a rolling boxcar for the 220 ms. Since we repeated the Wilcoxon rank-sum test 119 times, we applied the Bonferroni correction, so the significant level was *p* < 4.2 × 10^−4^ (=0.05/119). We marked the times at which these values showed significant differences (each ms) for each neuron (Fig. 3*D*, green horizontal line). To summarize these findings, we designated time periods showing significant differences across all 58 neurons, and made a histogram (significant time histogram, STH; 1 bin = 1 ms) of the percentage of recorded neurons showing significant differences in activity (Fig. 3*E*).

**Figure 4.**
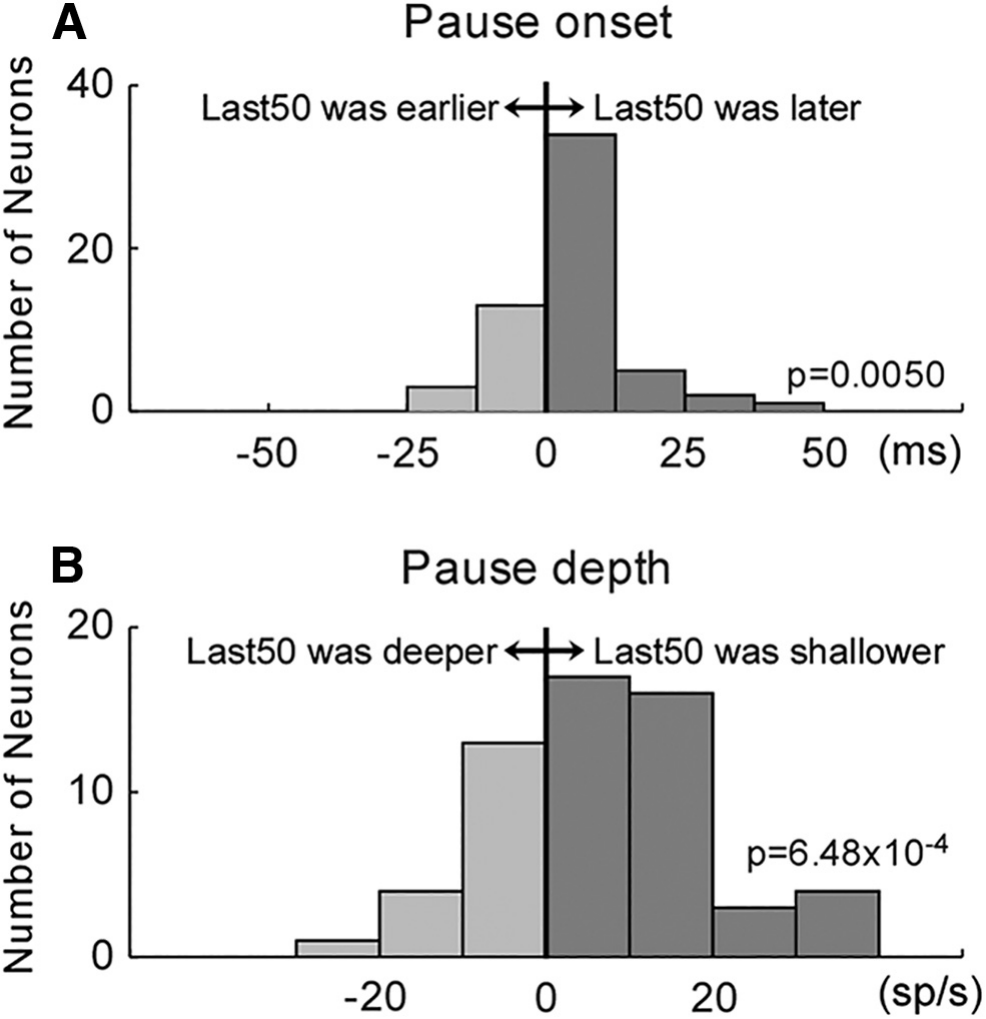
Change in the pause onset and depth. ***A***, Pause onset change. Positive values (*x*-axis): pause onset was later in the last 50 trials than in the first 50 trials. Significantly more neurons exhibited a positive value (*p* = 0.005, Wilcoxon signed-rank test). ***B***, Pause depth change. Positive values (*x*-axis): pause depth was shallower in the last 50 trials than in the first 50 trials. Significantly more neurons exhibited a positive value (*p* = 6.48 × 10^−4^, Wilcoxon signed-rank test).

To detect pause onset, we first calculated a derivative of the average SDF. From the time of the negative peak of the derivative, we searched backward to when the derivative crossed 0, and we marked this point as the pause onset (Fig. 3*D*, red and blue vertical broken lines, Fig. 4). The pause depth was determined by when the average SDF was the lowest.

**Figure 5.**
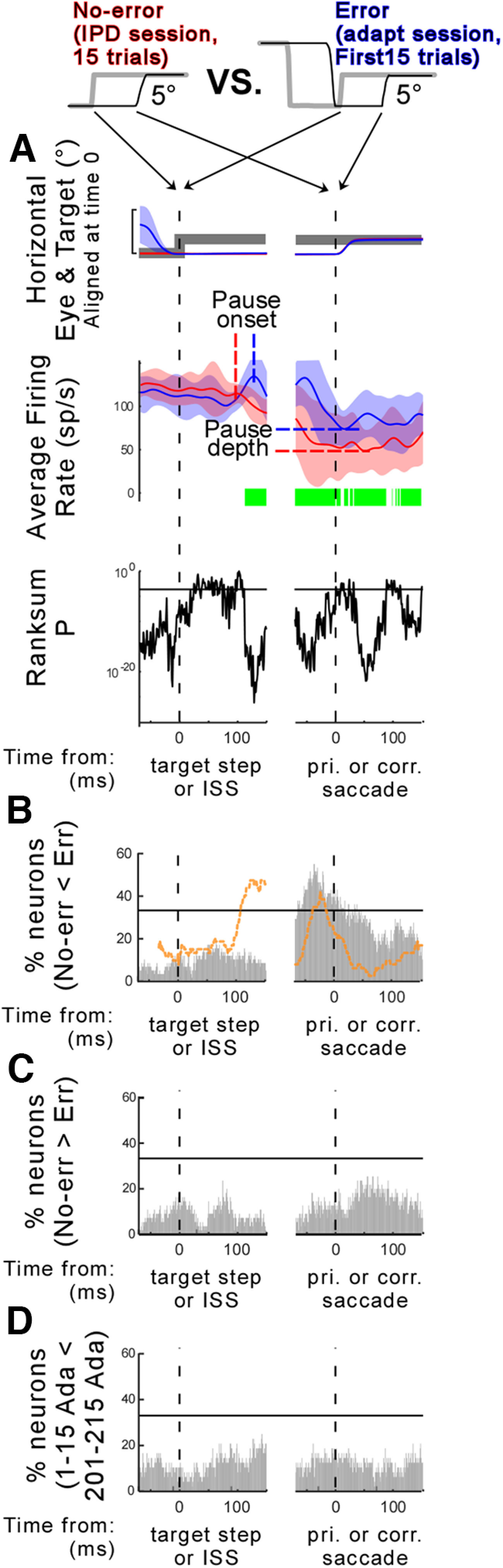
Comparison of the no-error activity and the postsaccadic visual error activity in SNr neurons. ***A***, Activity of a representative neuron (neuron #34, monkey E). Average firing rates of 15 trials associated with the 5° no-error (red) and 5°error (blue) trials. Data are aligned on the primary target step or ISS time (left), and primary saccade or corrective saccade onset (right). Top, middle, and bottom panels, The same organization as in Figure 3*D*. ***B***, ***C***, Summary of 58 neurons. The same organization as in Figure 3*E*, bottom panel, and *F*. Orange dashed line is a copy of the STH in Figure 3*E*. For each monkey, see Extended Data Figure 5-1. ***D***, Comparison of error trials between trial numbers 1–15 and 201–215 during adaptation session. Same organization as in ***B***.

**Figure 6.**
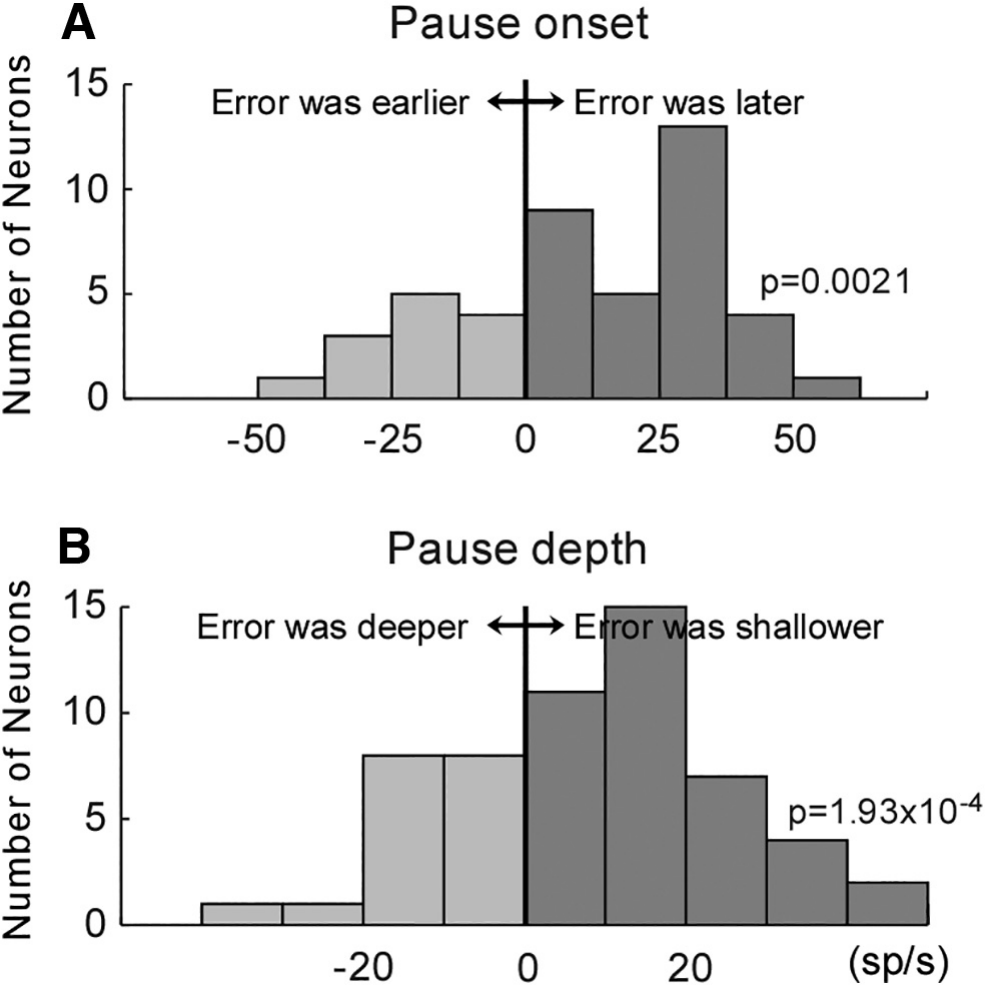
The difference in the pause onset and depth between error and no-error trials. ***A***, Difference in the pause onset. Positive values (*x*-axis): pause onset was later in the error trials than in the no-error trials. Significantly more neurons exhibited a positive value (*p* = 0.0021, Wilcoxon signed-rank test). ***B***, Difference in the pause depth. Positive values (*x*-axis): pause depth was shallower in the error trials than in the no-error trials. Significantly more neurons exhibited a positive value (*p* = 1.93 × 10^−4^, Wilcoxon signed-rank test).

We compared the SNr activity associated with the error and no-error trials. For the error trials, we took the first 15 saccades from the adaptation. Because the error size was 5° in the preferred direction, we took data from 5° target steps in the preferred direction for trials from the IPD session (Fig. 1*A*). If there were >15 such trials in the IPD session, we took the last 15 trials to minimize the sampling time difference between error and no-error trials. We then marked the significantly different times in each neuron. To use the same level of statistical significance that was employed for the adaptation session (comparison of 50 trials), we randomly sampled one trial from the 15 trials 50 times. We did the same random sampling for the comparison of the first 1–15 and 201–215 adaptation trials as a control of the sampling time difference (Fig. 5*D*). We computed the STH as a percentage of the 58 recorded neurons (e.g., % neurons; Fig. 5). We also compared the pause onset and pause depth for the error and no error trials (Fig. 6).

10.1523/ENEURO.0092-23.2023.f5-1 Extended Data Figure 5-1 STH of the percentage of recorded neurons (% neurons) that exhibited significantly greater activity in the error trials than in the no-error trials (Fig. 5*B*) for each monkey. The horizontal line indicates the threshold for significance (36.4% for monkey E and 29.7% for monkey Z, which is the mean ± 1 SD of all bins). Download Figure 5-1, TIF file.

**Figure 7.**
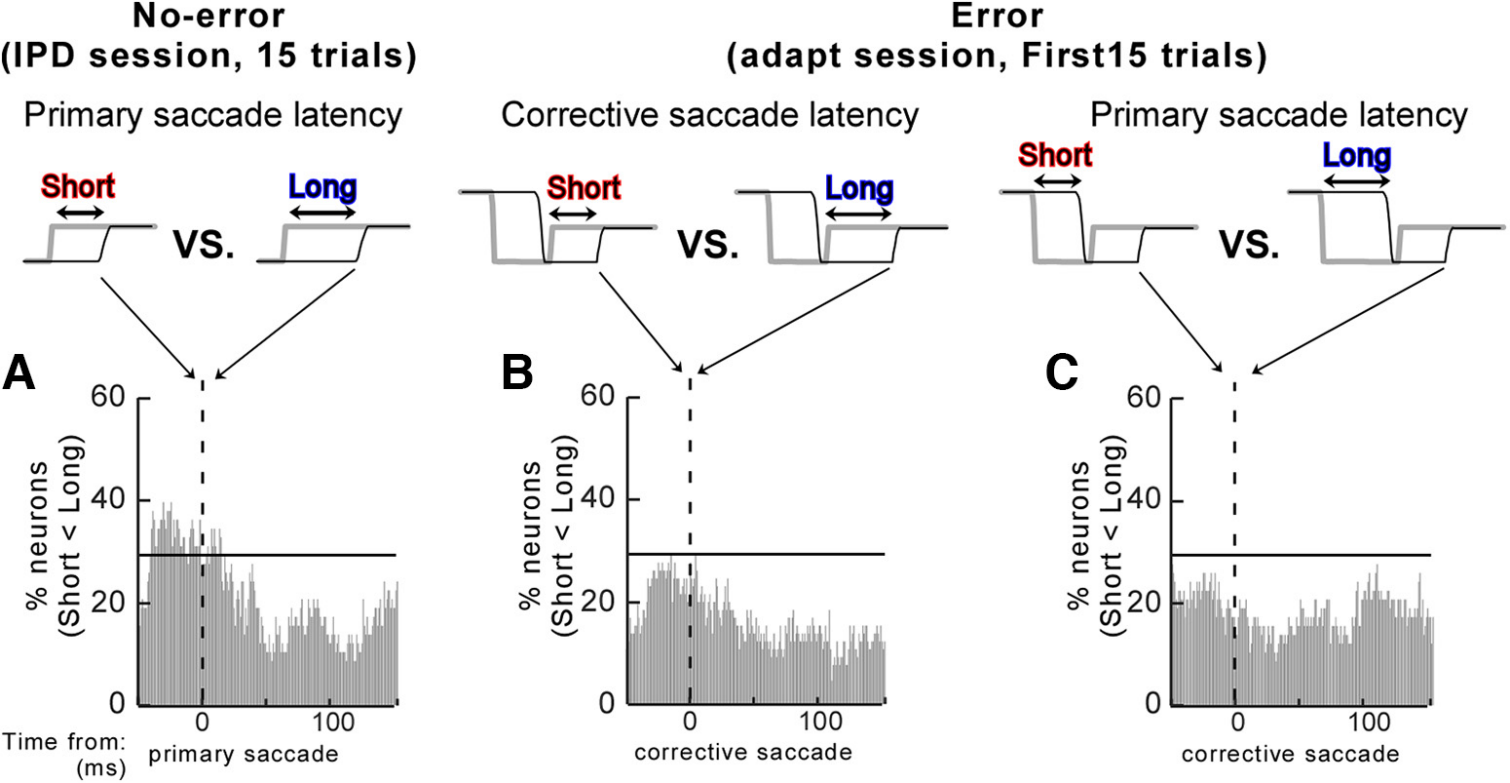
Comparison of the SNr activity between saccades with short and long latencies. ***A***, comparison of the no-error trials between saccades with short and long latencies. ***B***, ***C***, comparison of the error trials between corrective saccades (***B***) and primary saccades (***C***) with short and long latencies. Same organization as in Figure 5*A*,*B*. For each monkey, see Extended Data Figure 7-1.

To compare the SNr activity between short and long latency saccades in the 15 trials (Fig. 7), we split the 15 trials into two groups, that is, those with shorter or longer saccade initiations than the median latency of the 15 trials. We then marked the significantly different times in each neuron. Finally, we randomly sampled 50 times to use the same statistical test and established the STH of the percentage of 58 recorded neurons for each group.

10.1523/ENEURO.0092-23.2023.f7-1 Extended Data Figure 7-1 STH of the percentage of recorded neurons (% neurons) that exhibited significantly greater activity in the longer latency saccades than in the shorter latency saccades (Fig. 7) for each monkey. The horizontal line indicates the threshold for significance (28.6% for monkey E and 36.4% for monkey Z, which is the mean ± 1 SD of all bins). Download Figure 7-1, TIF file.

**Figure 8.**
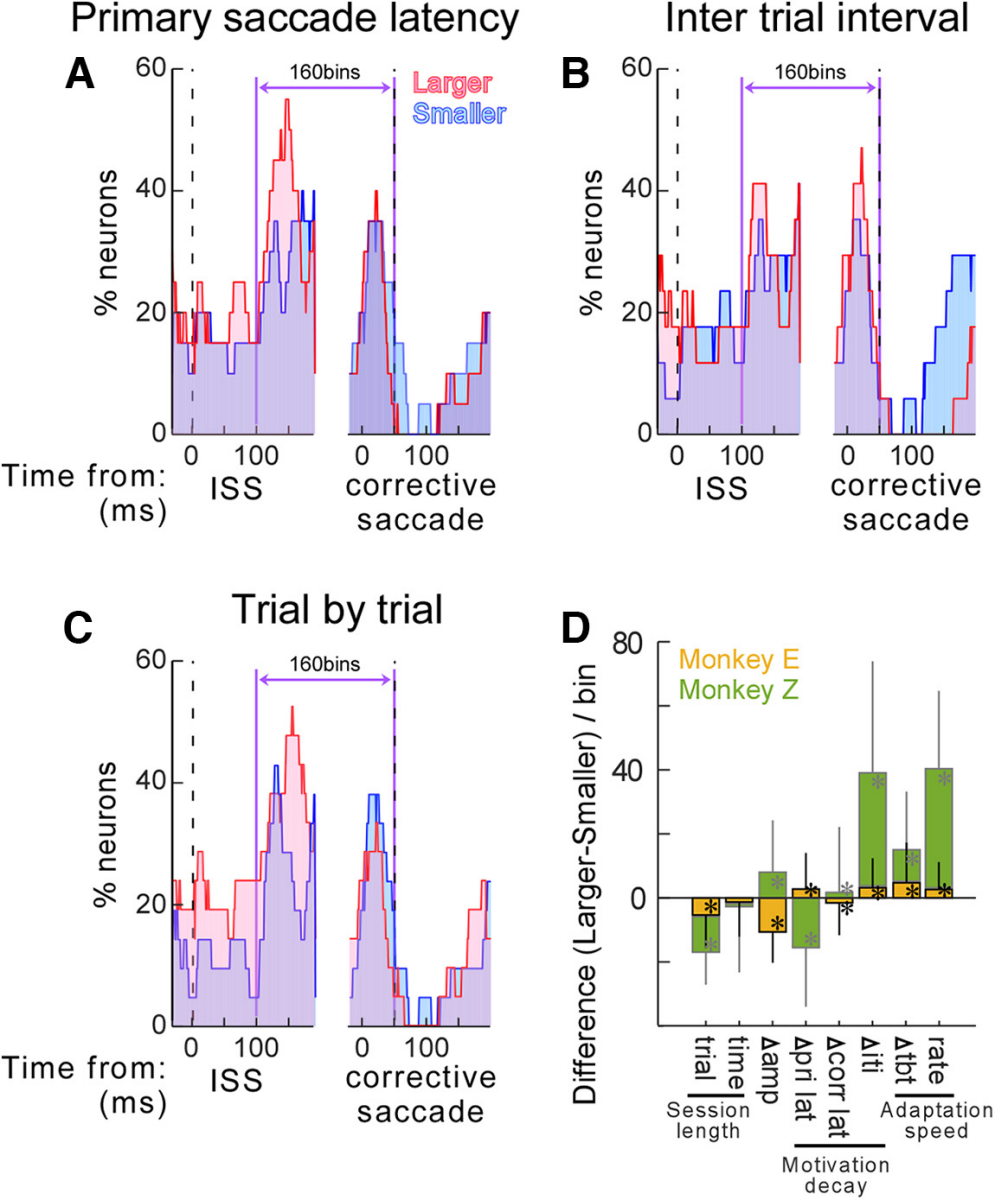
Comparison of the STH between a variety of parameters during adaptation. ***A–C***, STH of experiments with larger (pink) and smaller (blue) changes in the primary saccade latency (***A***), changes in the intertrial interval (***B***), and changes in trial-by-trial amplitude (***C***). Pink and blue STHs include 21 experiments from monkey E, respectively. The horizontal arrow between vertical lines indicates the 160 bins that showed an increase in the STH in Figure 3*E*. ***D***, The difference between the pink and blue STHs in the 160 bins. Yellow bars are monkey E, green bars are monkey Z. Trial: total trial numbers during adaptation session; time: total time during adaptation session; Δamp: amplitude change during adaptation session; Δpri lat: change in the primary saccade latency; Δcorr lat: change in the corrective saccade latency; Δiti: change in the inter trial interval; Δtbt: change in the trial-by-trial amplitude change; rate: rate constant of the adaptation course (outcome “b” of exponential fit; see Materials and Methods for the equation).

To examine the effect on SNr activity produced by other signals that were generated during the adaptation paradigm, we split the experiments performed by each monkey into two groups. For example, to examine the effect of the change in the primary saccade latency during the adaptation session, we sorted all experiments by the latency change and then split them into two groups; that is, the half with smaller values and the half with larger values. We then compared the percentage of neurons in each group present in the STH (Fig. 8).

## Results

The goal of this study was to characterize SNr activity during saccade adaptation to understand a possible neural mechanism that might influence superior colliculus (SC) activity during the postsaccadic error interval. In previous anatomic studies of nigrotectal projections in macaques (Jayaraman et al., 1977), the distribution of labeled terminals was only illustrated in the caudal 2/3 of the SC, where neurons with larger preferred vectors lie. However, the amplitude of postsaccadic error is usually small, for example, we used 5° in this study. Therefore, we thought it would be prudent to confirm the presence of anatomic connections between the SNr and rostral SC, where neurons with such smaller preferred vectors lie.

We injected biotinylated dextran amine (BDA) at physiologically specified locations in the rostral SC of monkey E and a third monkey (monkey N). The two injection experiments showed similar patterns of labeling. Figure 2*A* shows the location of the injection in monkey N. Micro-stimulation through an injectrode before an injection into the left rostral SC evoked a 4.1° saccade to the right and down (at 337°). Figure 2*B–D* shows BDA labeled neurons (red arrows) with black chromagen granules in the rostral lateral portion of the ipsilateral SNr. Thus, we demonstrated that SNr neurons project to the rostral, small saccade portion of the SC in the macaque.

Figure 3 documents a representative SNr neuron’s activity during amplitude decrease adaptation, which was induced by a visual error in the neuron’s preferred direction during primary saccades in the nonpreferred direction. Saccade amplitude decreased exponentially across the adaption trial series (Fig. 3*A*). Figure 3*B* shows the neuron’s activity aligned on the occurrence of the ISS, that is, primary saccade end (time 0; see also Fig. 3*C*). White dots represent the onset of each corrective saccade. This neuron did not pause for the primary saccades, as they were in their nonpreferred direction (before time 0), and did pause for the visual errors in the preferred direction (time ∼110 ms). This was an advantage of the amplitude decrease adaptation because the pause associated with the error was isolated, that is, there was no phasic activity before the ISS, so it allowed us to analyze the pause associated with the error. The pause gradually became shallower during adaptation. To document this change, we compared the first and last 50 trials (highlighted by red and blue lines next to the *y*-axis, respectively, in Fig. 3*B*). In Figure 3*D*, the middle panels compare the average firing rate associated with the first (red) and last (blue) 50 saccades during adaptation. In Figure 3*D*, the bottom panels show the *p* value of a Wilcoxon rank-sum test (see Materials and Methods) to define when in the trial the firing rate was significantly modified. Specifically, data in Figure 3*D*, left, show the primary saccade interval, which is aligned on the primary target step onset to display the baseline (before the target step) and visual activity preceding primary saccades (after the target step). There was no significant difference in the average firing rate. Data in Figure 3*D*, second from the left, show the primary saccade interval, which is aligned on the primary saccade onset to display the saccade related activity. Still, there was no significant difference in the average firing rate. Data in Figure 3*D*, second from the right, were aligned on the ISS to display the activity for the postsaccadic visual error interval. The averaged SNr activity associated with the last 50 saccades was significantly greater than that of the first 50 saccades at ∼100 ms (green horizontal line). The data for the corrective saccade interval also showed greater activity before the corrective saccade onset (Fig. 3*D*, right, green horizontal line).

To summarize the behavior of the 58 SNr neurons, we show the times when the differences in values were significant for all 58 neurons (Fig. 3*E*, top), along with a histogram (significant time histogram, STH) demonstrating the percentage of neurons showing significantly increased activity in the data for the postsaccadic error interval and corrective saccade interval (Fig. 3*E*, bottom; see also Extended Data Fig. 3-1 for each monkey). To detect the timing of this increase, we set a threshold, which is the mean (16.5%) ± 1 SD (9.3%) of STHs of all four intervals (= 25.8%; horizontal black line on the STH). The STH for activity increases rose above the threshold at 100 ms after the ISS and fell below the threshold at the corrective saccade onset (vertical purple lines). Figure 3*F* shows the percentage of recorded neurons which exhibit significantly less activity in the last 50 trials than in the first 50 trials. This value never passed the 25.8% threshold. Thus, SNr activity associated with the postsaccadic error only increased significantly during the adaptation session.

The increase of the SNr activity was accomplished by a change in the pause onset and in its depth. In the representative neuron shown in Figure 3*D*, the pause onset for the last 50 trials was later than that of the first 50 trials (blue and red vertical broken lines, respectively). The pause depth was shallower in the last 50 trials than in the first 50 trials (blue and red horizontal broken lines in the top row, second from right).

To summarize the pause onset of the 58 SNr neurons, we calculated the difference in the pause onset time between the first and last 50 trials. Positive values indicate the pause onset was later in the last 50 trials than in the first 50 trials (Fig. 4*A*, *x*-axis). Significantly more neurons exhibited later pause onsets in the last 50 trials than in the first 50 trials (*p* = 0.005, Wilcoxon signed-rank test). Figure 4*B* summarizes the difference in pause depth. Positive values indicate the pause depth was shallower in the last 50 trials than in the first 50 trials (Fig. 4*B*, *x*-axis). Significantly more neurons exhibited shallower pause depths in the last 50 trials than in the first 50 trials (*p* = 6.48 × 10^−4^, Wilcoxon signed-rank test). Thus, the pause started later and became shallower during the adaptation session, as the SNr activity increased.

Is the SNr activity associated with the postsaccadic visual error different from the activity that is not associated with the error? To address this question, we calculated the average firing rate of the visual error interval and of the corrective saccade interval for the first 15 trials during the adaptation session for each neuron. These data were classified as belonging to error trials. For no-error trials, we calculated the average firing rate for the visual interval and saccade interval after the 5° target steps into the neuron’s preferred direction. These data were collected for each neuron during the IPD session (Fig. 1*A*). Figure 5*A* documents a representative SNr neuron’s activity. Both trials had no preceding pause until ∼100 after the target step. The error trials exhibited significantly greater activity (shallower pauses) than the no-error trials for most of the time in the period after 100 ms from the ISS (Fig. 5*A*, middle panel, green lines).

Figure 5*B* is the STH that shows the percentage of the 58 recorded neurons that showed significant activity differences (see also Extended Data Fig. 5-1 for each monkey). We set a threshold, which is the mean (20.2%) ± 1 SD (13.1%) of STHs of two intervals (=33.3%; horizontal black line on the STH). These values did not pass the threshold in the data aligned on the ISS, but they passed it in the data aligned on the corrective saccade. As a reference, the STH of the first and last 50 adaptation trials in Figure 3*E* was overlaid (orange dashed line). During the corrective saccade interval, more neurons exhibited greater activity in the error versus no-error comparison than in the first 50 trials versus the last 50 trials comparison.

Figure 5*C* shows the percentage of recorded neurons that exhibit significantly less activity in the error trials than in no-error trials. These values never passed the 33.3% threshold. Because error trials took place ∼10 min after no-error trials (Fig. 1*A*), there is a possibility that the increase in the percentage of recorded neurons showing effects might reflect the time of their recording rather than the error versus no-error difference. To control for this, we compared the first 1–15 adaptation trials with trials 201–215 that came ∼10 min after the first trial (Fig. 5*D*). The significance of the difference between these values still never passed the 33.3% threshold, indicating that time, per se, cannot explain the increase.

Figure 6 compares the pause onset and pause depth values for the error and no-error trials. Positive values indicate the pause onset was later (Fig. 6*A*, *x*-axis) and the pause depth was shallower (Fig. 6*B*, *x*-axis) in the error trials than in the no-error trials. Significantly more neurons exhibited later pause onsets (Fig. 6*A*) and shallower pauses (Fig. 6*B*) in the error trials than in the no-error trials (onset: *p* = 0.0021, depth: *p* = 1.93 × 10^−4^, Wilcoxon signed-rank test). Thus, the pauses associated with the errors were significantly different from the pauses that were not associated with the errors.

It is known that SNr activity is stronger (= shallower pause) for longer latency saccades than for shorter latency saccades (Hikosaka et al., 2000; Yasuda et al., 2012). We examined this feature with our data. Figure 7*A* shows this comparison for the no-error trials. We set a threshold, which is the mean (20.2%) ± 1 SD (8.9%) of STHs (= 29.1%; horizontal black line on the STH). The data comparing longer and shorter saccades aligned on primary saccade onset passed the threshold for the period starting ∼40 ms before the saccade until ∼15 ms after the saccade. For the error trials, we compared two saccade latencies: those of corrective saccades (Fig. 7*B*) and those of primary saccades (Fig. 7*C*). In both comparisons, the STH values did not reach the 29.1% threshold (see also Extended Data Fig. 7-1 for each monkey). Thus, the stronger SNr activity for longer latency saccades was detected in no-error trials, which is consistent with previous studies, but it was not detected in error trials, suggesting that saccade latency did not have a clear effect on the SNr activity associated with the postsaccadic error.

Many parameters change during saccade adaptation. Furthermore, they vary between experiments. Table 1 summarizes the mean and 1 SD of a number of parameters across 58 experiments. Taking advantage of this variability, we examined the correlation of these parameters with the SNr activity increase during adaptation.

Figure 8*A* compares the experiments from monkey E that displayed larger or smaller changes in primary saccade latency during adaptation. The larger or smaller change groups each contain half of the monkey E’s experiments (*n* = 21). The STH was higher in the larger change group (pink) than the smaller change group (blue). We compared the 160 bins that occur between 100 ms after the ISS to the onset of the corrective saccade. These were all bins in which the comparison of the STH of the first and last 50 saccades passed the threshold (Fig. 3*E*, purple vertical lines). The mean difference (per bin) was 2.78 ± 11.3 (*p* = 9.81 × 10^−3^, Wilcoxon signed-rank test; Fig. 8*D*, yellow bar at “Δ pri lat”), indicating that more neurons in the larger group exhibited an increase in the activity during adaptation. This positive value supports the possibility that the change in the primary saccade latency correlates to the increase in the SNr activity. However, monkey Z showed a negative value (mean difference = −15.5 ± 18.5, Wilcoxon signed-rank test *p* = 3.48 × 10^−16^; Fig. 8*D*, green bar at “Δ pri lat”). Because of this discrepancy, we could not come to any conclusion with respect to primary saccade latency effects. For another indicator of motivation decay during the adaptation session, we examined change in corrective saccade latency. A similar discrepancy between the correlations in the two animals was found (Fig. 8*D*, yellow and green bars at “Δ corr lat”) so again no conclusion could be reached. Finally, we examined change in the intertrial interval (Fig. 8*B*). Both monkeys exhibited positive values (Fig. 8*D*, yellow and green bars at “Δ iti”). This supports the possibility that the change in the intertrial interval, perhaps because of a change in motivation, correlates with the increase in SNr activity.

Figure 8*C* displays change in trial-by-trial amplitude. More neurons in the larger change group exhibited an increase in activity during adaptation than those in the smaller change group. Both monkeys exhibited positive values (Fig. 8*D*, yellow and green bars at “Δ tbt”). For another indicator of adaptation speed, we examined the rate constant of the exponential fit. Again, both monkeys exhibited positive values (Fig. 8*D*, yellow and green bars at “rate”). Thus, these data suggest that the change in the SNr activity is correlated with the slowing of adaptation speed.

Another factor that was examined was adaptation session length. In this case, we examined the total number of trials during the session. Both monkeys exhibited negative values (Fig. 8*D*, yellow and green bars at “trial”), so this correlation cannot explain the SNr activity increase. We also examined the time duration of the adaptation session. In this case, there was no significant difference related to session duration (Fig. 8*D*, yellow and green bars at “time”; *p* = 0.16 for monkey E and 0.78 for monkey Z, Wilcoxon signed-rank test). Finally, we examined the total amplitude change during the session. Once again, a monkey-to-monkey discrepancy was found (Fig. 8*D*, yellow and green bars at “Δ amp”) precluding a conclusion with respect to this variable.

## Discussion

We asked two initial questions in this study. (1) Whether the SNr activity changes during adaptation. (2) If so, whether the SNr activity associated with the postsaccadic error increases. The answers to both these questions were yes; the pause associated with the error became shallower and the onset was delayed as the trials in a session proceeded (Figs. 3, 4). In addition, the SNr activity associated with the error trials had different features than the activity for trials which were not associated with the error. The pause for the error trials was shallower and the onset was later than for the no-error trials (Figs. 5, 6). The pause for error trials was not correlated to the reaction time, while the pause for no-error trials was correlated to the reaction time (Fig. 7). Finally, we asked a third question. (3) “What parameters of the adaptation paradigm might influence the SNr increase?” This question could only be partially answered because, as noted in the Introduction, we lacked information on many of the variables and did not establish cause and effect in this study. Nevertheless, we examined the parameters which were available in our data. The visual stimulus for the primary saccade was constant during the entire adaptation session, so this parameter can be excluded. Primary saccade amplitude decreases and latency increases had no consistent correlation (Fig. 8), so these parameters are inconclusive with respect to influencing SNr activity. The postsaccadic visual error amplitude and the corrective saccade amplitude were constant during the entire adaptation session (Table 1), so these can be excluded. The corrective saccade latency increase was not consistently correlated with SNr activity (Fig. 8), so this variable is inconclusive with respect to influencing SNr error trial changes. We examined adaptation speed in two different ways, trial-by-trial amplitude change and rate constant of exponential fit. Both showed a positive correlation. This suggests that SNr activity is related to adaptation speed. We also examined aspects of repetition. The total number of trials and the time of the adaptation session had no positive correlation (Fig. 8*B*), so these are less likely to influence the pause. The motivation decay that occurs across sessions was measured in three different ways, by comparison of: the increase in the latency of the primary saccade, the latency of the corrective saccade, and the intertrial interval. All were significantly increased during adaptation (Table 1). The comparison of primary and corrective saccade latencies was inconclusive (Fig. 8), which makes sense because the latency did not correlate with the pause for the error trials (Fig. 7). However, the intertrial interval showed a positive correlation. This suggests that the motivational decay during the adaptation session might influence the SNr activity associated with the error. Taken together, the SNr activity associated with the postsaccadic error increased during adaptation. This is consistent with the decrease in SC activity, and it was somewhat correlated to motivation and adaptation speed (discussed below).

It should be noted that the change in the pause in neurons was idiosyncratically different from neuron to neuron. One reason might be the designation of the preferred direction. Because the tuning of the SNr is very broad (Fig. 1*B*), the designation was somewhat arbitrary. Consequently, the difference might mask a weak correlation of the parameters. Therefore, this analysis does not eliminate the possibility of some influence by parameters for which we did not detect a correlation. For example, the change in the SNr activity during adaptation (Fig. 8) was not consistently correlated to the saccade latency, which can represent motivation. However, it has been shown that when the reaction time is controlled by reward motivation, saccades with shorter reaction times exhibit faster adaptation (Kojima and Soetedjo, 2017b). Furthermore, the inactivation of the SNr shortens the latency and facilitates adaptation (Kojima and May, 2021). These studies suggest that latency and/or motivation can affect saccade adaptation. Therefore, the present study should not be interpreted to conclude that saccade latency, SNr activity, and saccade adaptation are unrelated.

**Figure 9.**
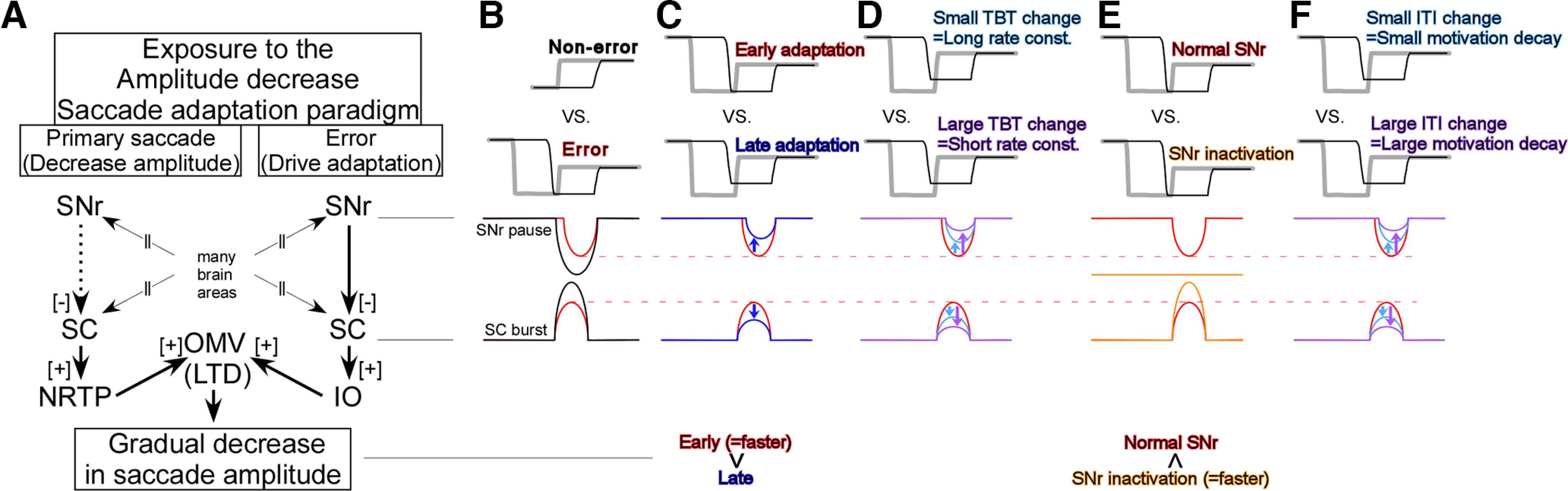
Schematic of the SNr, SC, to the oculomotor vermis (OMV) and underlying saccade adaptation effects. ***A***, Connections from the SNr to the OMV. SNr, SC, and NRTP (nucleus reticularis tegmenti pontis) under the “Primary saccade” column are related to the primary saccade, SNr, SC, and IO (inferior olive) under the “Error” column are related to the error signal. The error signal induces long-term depression (LTD) in the OMV to reduce the primary saccade signal. As a result, the saccade amplitude is decreased gradually. ***B–F***, SNr and SC activity for; no-error (black) and error (red) trials (***B***; see Extended Data Fig. 9-1 for more details), early (red) and late (blue) adaptation trials (***C***), small trial-by-trial amplitude change (=long rate constant; light blue) and large trial-by-trial amplitude change (=short rate constant; purple) during adaptations (***D***), under normal condition (red) and SNr inactivation condition (orange; ***E***), small intertrial interval change (light blue) and large intertrial interval change (purple) during adaptations (***F***). Bottom: “faster,” Adaptation is faster in the early adaptation session than in the later adaptation, in the SNr inactivation condition than in the normal SNr condition.

During the amplitude decrease adaptation, the SNr that is responsible for the primary saccade is on the other side of the brainstem from the SNr that pauses for the error (Fig. 9*A*). This study did not examine the SNr activity for the primary saccade because the SC activity for the primary saccade does not change during adaptation (Frens and Van Opstal, 1997; Takeichi et al., 2007; Quessy et al., 2010). Previous studies showed that inactivation and micro-stimulation of the SNr affect the metrics (amplitude) of memory-guided saccades, however, these manipulations do not affect the metrics of visually-guided saccades (Hikosaka and Wurtz, 1985; Basso and Liu, 2007; Kojima and May, 2021). Also, visually-guided saccades are mostly preserved in Parkinson’s disease patients compared with the memory-guided saccades (Pretegiani and Optican, 2017), suggesting that the basal ganglia activity may not have as much effect on the metrics of visually-guided saccades. Note, however, that the basal ganglia can be involved in managing the triggering (latency) and dynamics (velocity profile) of visually-guided saccades. Thus, the change, if any, in the SNr activity responsible for the primary saccade probably does not play an important role in visually-guided saccade adaptation which modulates their metrics.

10.1523/ENEURO.0092-23.2023.f9-1 Extended Data Figure 9-1 Examples of the SC activity of visual (***A***, ***B***) and visuomotor (***C***, ***D***) neurons in error and no error trials. The optimal amplitudes for both neurons were ∼3°. Red: no error, blue: error, grey: target. ***A***, ***C***, Horizontal (top) and vertical (bottom) eye position aligned on the target step. ***B***, ***D***, Average discharge of the SDF. The visual activity in the error condition was smaller. Green arrows indicate the difference between error and no error conditions. The other 20 SC neurons recorded showed a similar reduction. Download Figure 9-1, TIF file.

The primary saccade signal from the SC is conveyed to the oculomotor vermis and induces a gradual decrease in simple spike activity during the adaptation [long-term depression (LTD); Fig. 9*A*; Catz et al., 2008; Kojima et al., 2010]. As a result, the primary saccade amplitude is decreased gradually. The LTD is driven by the error signal, which is represented as the complex spike activity in the oculomotor vermis (Catz et al., 2005; Soetedjo and Fuchs, 2006; Soetedjo et al., 2008a,b) and which originates from the SC (Kaku et al., 2009; Soetedjo et al., 2009; Kojima and Soetedjo, 2017a, 2018). The SC burst associated with the error is smaller than the activity that is not associated with the error (Fig. 9*B*; Extended Data Fig. 9-1). Consistent with this difference, the SNr pauses associated with the errors are shallower than the pauses which are not associated with the errors (Figs. 5, 9*B*). During adaptation, the SC burst associated with the error decreases (Fig. 9*C*; Kojima and Soetedjo, 2017a) and the SNr pause becomes shallower (Figs. 3, 9*C*). Thus, the shallower SNr pause likely contributes to the weaker SC burst.

Because this SC error signal drives adaptation, one might expect that stronger error signals induce stronger adaptation. The data support this expectation. First, the adaptation is stronger at the beginning than at the end of the adaptation session, as it follows an exponential time course (Fig. 3*A*). Similarly, the SNr pause for the error is deeper at the beginning, so the SC burst for the error is stronger (Fig. 9*C*). The stronger SC error signal drives the stronger (=faster) adaptation at the beginning of the error trial series. Second, when the change in the trial-by-trial amplitude change is smaller, which is equivalent to a long rate constant, the SC maintains the stronger error signal longer (Fig. 9*D*). The stronger SC error signal leads to a stronger adaptation. Third, SNr inactivation facilitates adaptation (Kojima and May, 2021). The inactivation eliminates SNr activity. As a consequence, the SC error signal is stronger (Fig. 9*E*). This stronger SC activity can, in turn, induce stronger adaptation. Thus, we speculate that weaker SNr activity (=deeper pause) allows the SC neurons to fire more strongly and assist in driving adaptation faster. Obviously, no-error trials do not induce adaptation, although the SNr activity is the weakest during these trials, and the SC activity is the strongest (Fig. 9*B*). This is simply because there is no preceding saccade to be adapted.

Finally, when the intertrial interval change is smaller, which implies less motivational decay during the adaptation session, the SNr activity increase is smaller, so the SC maintains the stronger error signal (Fig. 9*F*). The stronger SC error signal maintains the stronger adaptation. Taken together, we speculate that the SNr might act as a hub to connect motivation and adaptation. Motivation wanes with a long and repetitive task like a typical >2-h adaptation session. This gradual decay of motivation leads to extended reaction times and increased SNr activity. Because the SNr activity associated with error did not encode the trial-by-trial variability of the reaction time (Fig. 7), SNr activity is likely to represent motivation level, not trial-by-trial reaction time, per se.

Since there are a number of projections from and to the SC, many neural mechanisms might be involved in regulating the SC error sensitivity. This study simply suggests that the increase in the SNr activity is one of the mechanisms and it does not reject other possible mechanisms. This study does not, in itself, provide causal evidence that the SNr influences the downstream adaptation circuit. However, this relationship has been established by the combination of this and previous studies: (1) SNr projects to the rostral SC (Fig. 2); (2) an injection study has shown that inactivation of the SNr facilitates saccade adaptation (Kojima and May, 2021); (3) clinical studies have shown that the adaptation of Parkinson’s disease patients is slower than that of age matched control subjects (MacAskill et al., 2002; Abouaf et al., 2012), suggesting that the basal ganglia influence saccade adaptation. In summary, this study has fulfilled its intended examination of SNr activity under controlled physiological conditions to address whether it actually changes during adaptation and what aspects of the activity change.

### Anatomical findings

There is considerable evidence to suggest that the connectivity patterns of the rostral and caudal colliculus are not the same for many pathways (May et al., 1992; Olivier et al., 1998; Takahashi et al., 2005), making a definitive exploration of nigral input to the rostral SC necessary. In the only previous anterograde tracer study of nigrotectal projections in macaques (Jayaraman et al., 1977), the terminal label was only illustrated in the caudal 2/3 of the SC, where neurons with larger preferred vectors lie. Furthermore, previous retrograde studies in the macaque have used large injections of the SC, which often did not include its rostral pole (Beckstead and Frankfurter, 1982; Parent et al., 1983; Francois et al., 1984). Since both the primary saccades and the error signals used for adaptation in our study were fairly small, and small targets are coded within the rostral SC, we felt it was necessary to specifically examine the nigrotectal pathways from the rostral SC. The present study shows that a SNr input to the rostral SC is present in macaques. The injection sites for both animals were quite small (∼1.0 mm across) and were centered in the intermediate gray layer. So while they may have involved more than the locus of the vector identified by stimulation, they did not involve the caudally located large saccade portion of the SC. Ascending axons from the SNr travel directly dorsally and enter the SC at the level they terminate (May and Hall, 1986). Therefore, our rostral SC injections should not involve axons destined for the caudal SC by axon-of-passage uptake. Consequently, we are confident that the results shown here represent actual labeling from the injection sites. The pattern of labeled nigrotectal cells we observed fell within the previously reported distribution of monkey nigrotectal neurons as a whole (Beckstead and Frankfurter, 1982; Parent et al., 1983; Francois et al., 1984). We did not observe the bilateral projections to rostral SC reported for the galago (Huerta et al., 1991), but this may be because the BDA technique used here is less effective at labeling minor projections. In summary, we have shown here that a nigrotectal connection to the appropriate part of the SC to drive saccade adaptation is present.

## Synthesis

Reviewing Editor: David Schoppik, New York University Decisions are customarily a result of the Reviewing Editor and the peer reviewers coming together and discussing their recommendations until a consensus is reached. When revisions are invited, a fact-based synthesis statement explaining their decision and outlining what is needed to prepare a revision will be listed below. The following reviewer(s) agreed to reveal their identity: Neeraj Gandhi, Richard Krauzlis.

The reviewers and I agree: the manuscript is much improved, and while challenges remain in explaining all of the observations, the added analyses bolster the case for acceptance. The reviewers had a number of comments/suggestions to improve the clarity that I append below:

Both reviewers agreed that Figure 1 detracted from the manuscript. They found it hard to follow (as experts in the field) and suggested instead to treat it textually as the final paragraph of the instruction.

The reviewers found the text challenging to follow. We respectfully acknowledge the difficulty but it would be a shame to lose the readers after the hard work. Others in similar situations have used online tools like Grammarly or paid for professional copy-editing to allow the manuscript to shine as it deserves.

Analysis suggestions / questions

1) Threshold criterion. The authors arrive at 25.8% of the neurons for threshold criterion (Line 350). This is fine for the analysis at hand (Figure 4). But I was puzzled that they used the same criterion for subsequent analyses (e.g., Figures 6 and 8). Should they not compute the threshold specific to each analysis? If not, some justification is warranted.

2) Figure 9D highlights a number of inconsistencies between the two animals. This made me wonder why the core analyses (e.g., Figures 4, 6, and 8) were far more consistent. I came to notice that the data are heavily weighted by one animal (43 neurons from monkey E). Monkey Z contributed only 15 neurons. The authors therefore need to provide evidence that these results were consistent across the two animals.

3) We liked the structure of analyses reported in Figures 4 and 6.

Line-by-line notes

Line 9. Suggest replacing “it” with “SNr”.

Line 10. Sentence beginning “In fact, the SNr...” is confusing. Are you referring to something established in the literature, or to your results?

Line 46: ...the most “commonly” studied saccade adaptation...

Line 59. Perhaps: correlated with the motivational state

Line 77: Insert ‘?’ after trials.

Lines 93-94. Perhaps: centered on the midline to target both superior colliculi.

Line 136. Here and elsewhere (lines 145/149), “Fig 1” actually refers to “Fig 2”

Line 141: Please reference Fig 2C near here

Line 375. Here and elsewhere, “data” are plural (“These data were...”)
