# Supplementary figures and images for: Activity of the Substantia Nigra Pars Reticulata during Saccade Adaptation
Source: eNeuro. 2023 Sep 11;10(9):ENEURO.0092-23.2023. doi: 10.1523/ENEURO.0092-23.2023 (PMC10500979; doi:10.1523/ENEURO.0092-23.2023)

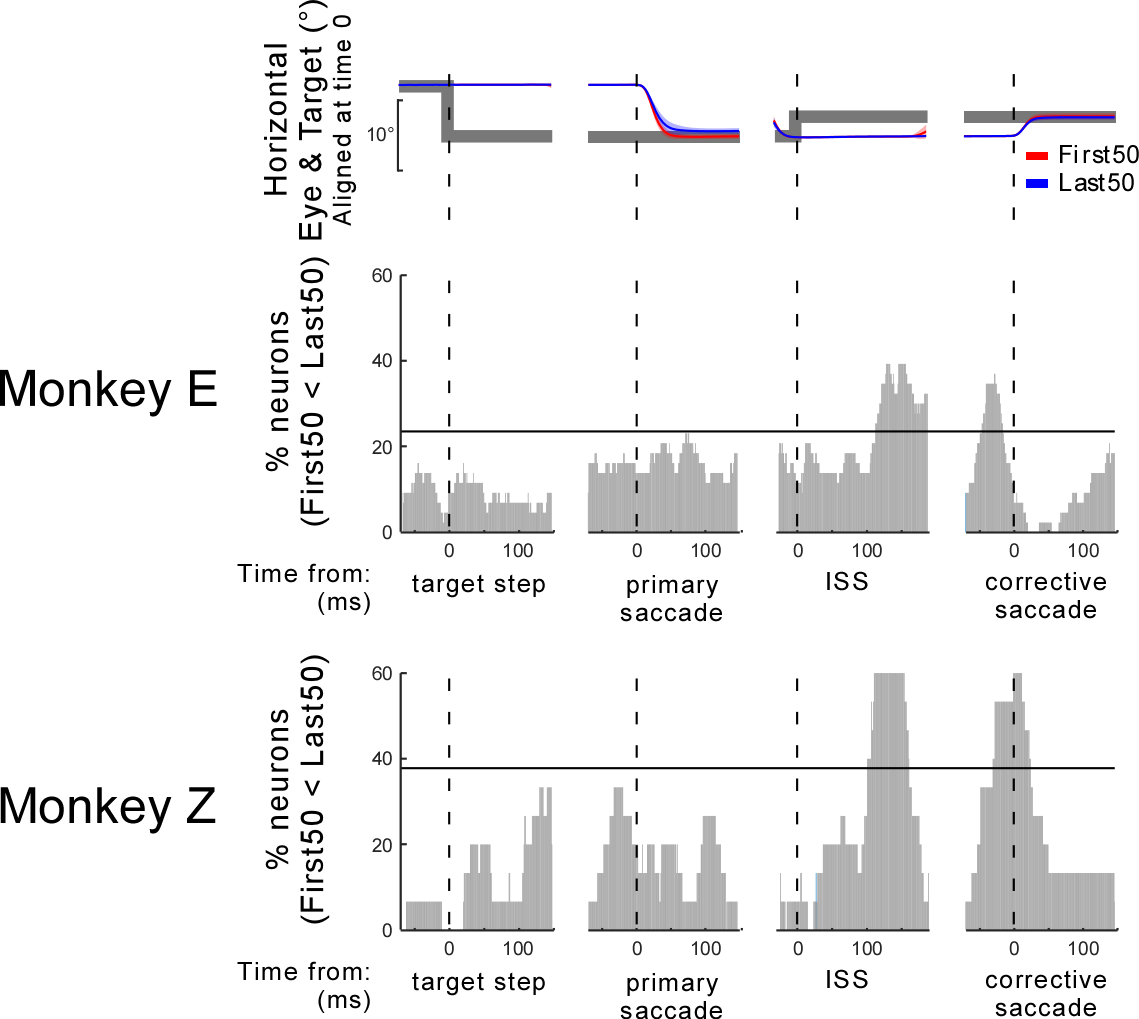

Supplement: Extended Data Figure 3-1 — STH of the percentage of recorded neurons (% neurons) that exhibited significantly greater activity in the last 50 saccades than in the first 50 saccades (Fig. 3E, bottom) for each monkey. The horizontal line indicates the threshold for significance (23.7% for monkey E and 37.8% for monkey Z, which is the mean ± 1 SD of all bins). Download Figure 3-1, TIF file. [file enu-eN-NWR-0092-23-s01.tif]

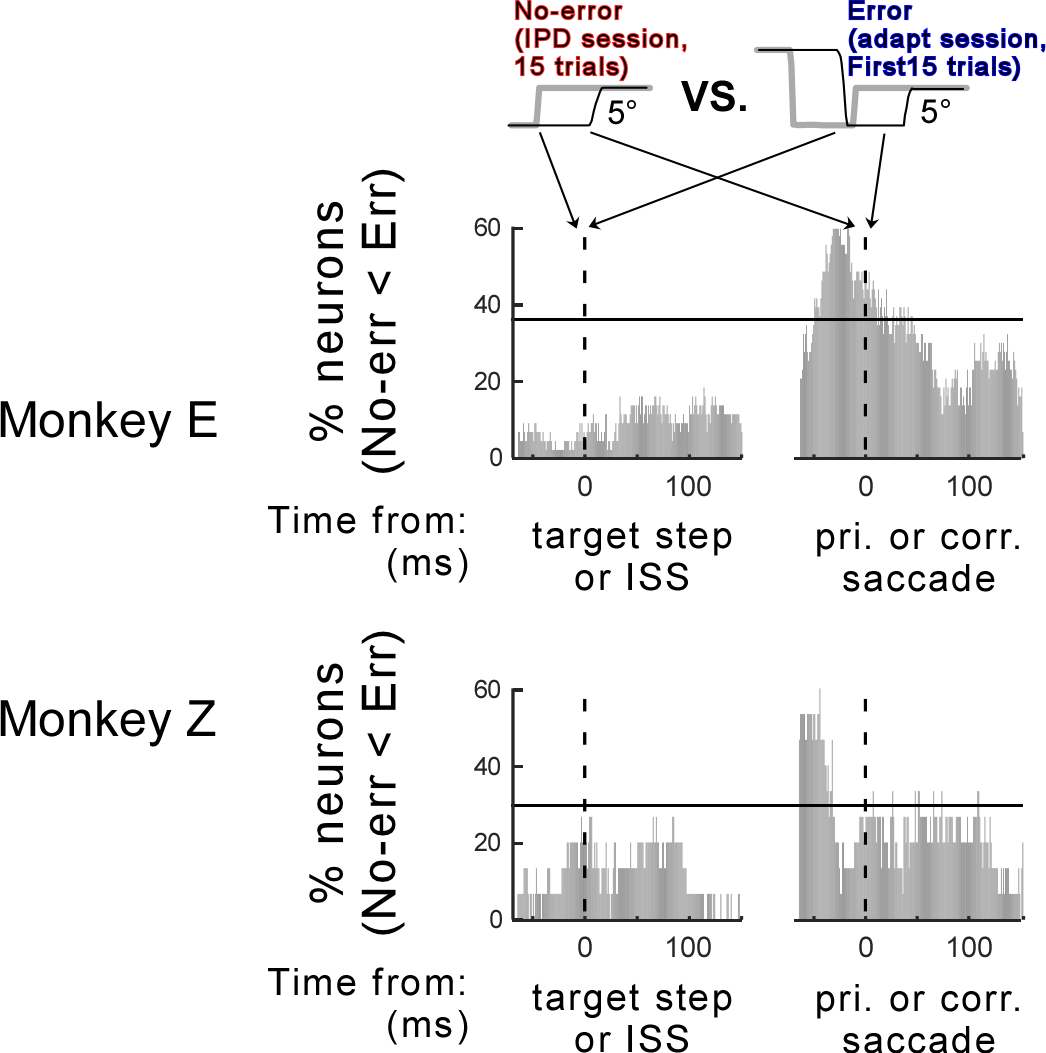

Supplement: Extended Data Figure 5-1 — STH of the percentage of recorded neurons (% neurons) that exhibited significantly greater activity in the error trials than in the no-error trials (Fig. 5B) for each monkey. The horizontal line indicates the threshold for significance (36.4% for monkey E and 29.7% for monkey Z, which is the mean ± 1 SD of all bins). Download Figure 5-1, TIF file. [file enu-eN-NWR-0092-23-s02.tif]

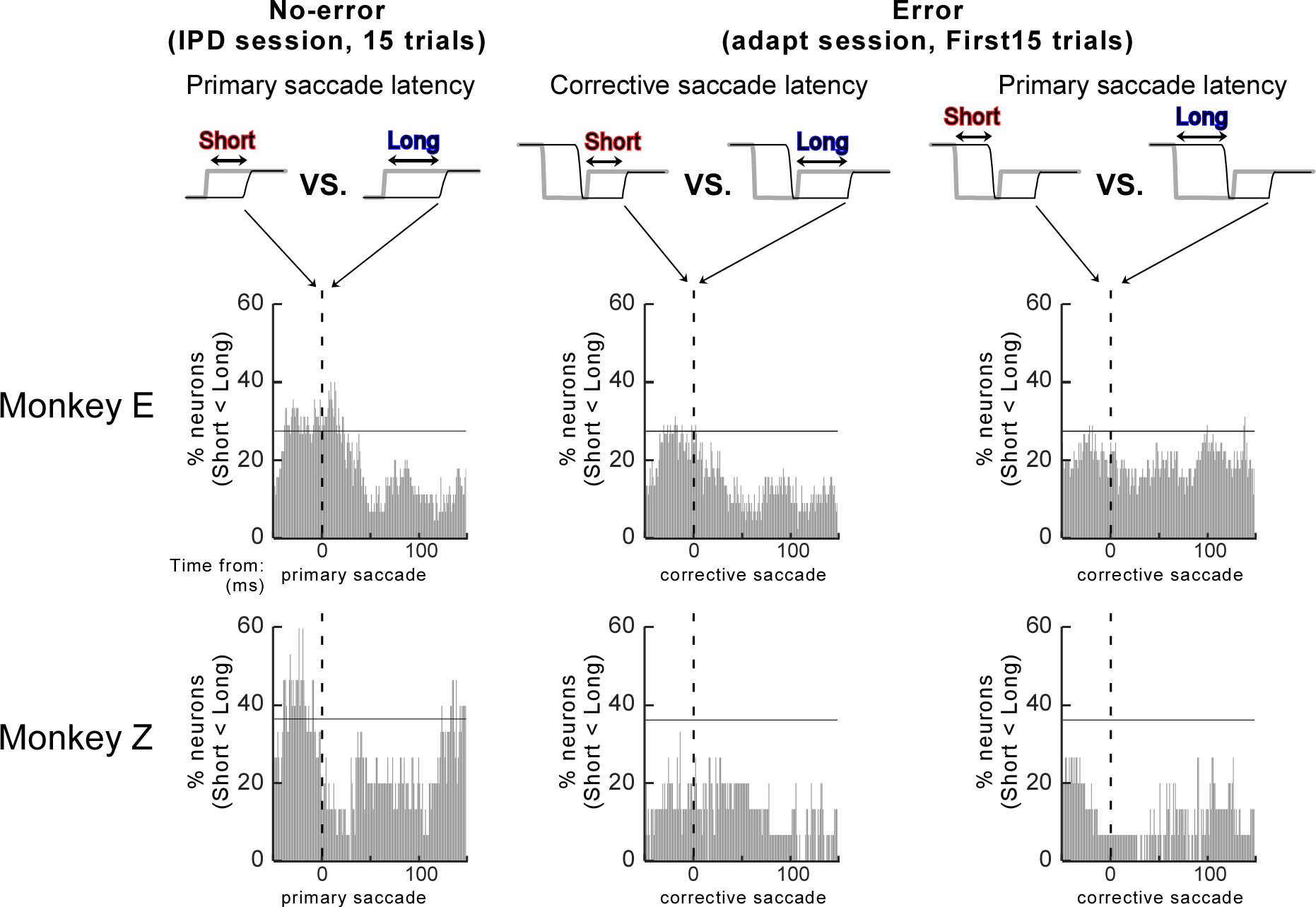

Supplement: Extended Data Figure 7-1 — STH of the percentage of recorded neurons (% neurons) that exhibited significantly greater activity in the longer latency saccades than in the shorter latency saccades (Fig. 7) for each monkey. The horizontal line indicates the threshold for significance (28.6% for monkey E and 36.4% for monkey Z, which is the mean ± 1 SD of all bins). Download Figure 7-1, TIF file. [file enu-eN-NWR-0092-23-s03.tif]

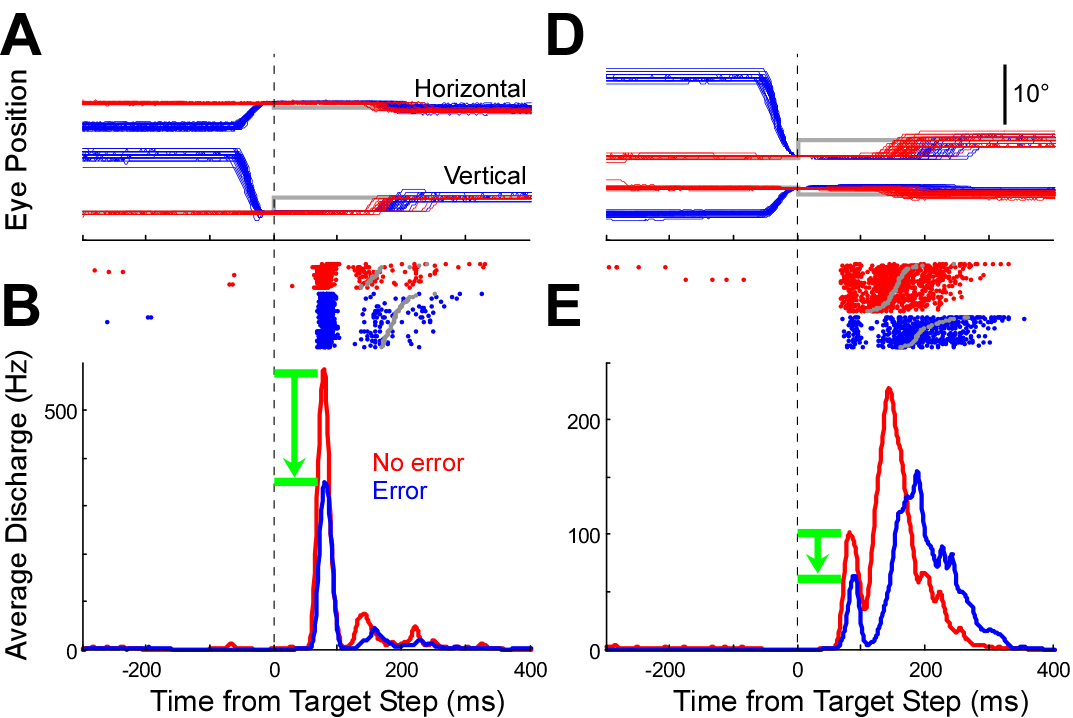

Supplement: Extended Data Figure 9-1 — Examples of the SC activity of visual (A, B) and visuomotor (C, D) neurons in error and no error trials. The optimal amplitudes for both neurons were ∼3°. Red: no error, blue: error, grey: target. A, C, Horizontal (top) and vertical (bottom) eye position aligned on the target step. B, D, Average discharge of the SDF. The visual activity in the error condition was smaller. Green arrows indicate the difference between error and no error conditions. The other 20 SC neurons recorded showed a similar reduction. Download Figure 9-1, TIF file. [file enu-eN-NWR-0092-23-s04.tif]
